# ATF5: development of oncogenic resistance to radiotherapy

**DOI:** 10.18632/aging.100775

**Published:** 2015-07-07

**Authors:** Seiichiro Ishihara, Hisashi Haga

**Affiliations:** Faculty of Advanced Life Science, Hokkaido University, Kita-ku, Sapporo 060–0810, Japan

Radiotherapy involves killing of cancer cells with irradiation and is a widely accepted therapeutic method for treating various types of cancers. However, a portion of cancer cells survives even after radiotherapy and leads to the development of recurrent tumors that gives rise to more malignant phenotypes than that before irradiation. We previously reported that any lung cancer cells that survive after irradiation have high invasiveness, which is regulated by several molecules, such as integrin β1 [1], myosin regulatory light chain (MRLC) [2], epidermal growth factor receptor [3], and filamin B [4]. These molecules play important roles in cell migration. However, the ability of lung cancer cells to survive irradiation and develop aggressive tumors after irradiation is poorly understood.

We recently reported that activating transcription factor 5 (ATF5) leads to the development of radioresistance and malignant phenotypes in lung cancer cells after irradiation [5]. The two functions of ATF5 demonstrate “oncogenic resistance,” which implies that several molecules show enhanced resistance against some therapeutic techniques and simultaneously trigger cancer progression [6]. We hypothesize that ATF5 is one of the key molecules involved in development of oncogenic resistance to radiotherapy.

First, we revealed that ATF5 enhances radioresistance in lung cancer cells. A549 human lung cancer cells that overexpress ATF5 shows higher survival ability after 10 Gy irradiation than that of the control A549 cells. Moreover, ATF5 expression and radioresistance of A549 cells varies depending on the cell cycle. During the G1-S phases of the cycle and not during the G2-M phases, A549 cells show high expression of ATF5 and radioresistance. We also demonstrated that ATF5 enhances radioresistance by promoting G1/S transition in the cell cycle. Thus, ATF5 plays an important role in radioresistance via regulation of the cell cycle in lung cancer cells.

In addition, we found that ATF5 triggers lung cancer progression. ATF5 overexpression enhanced the proliferative and invasive abilities of A549 cells in *in vitro* cultures on a three-dimensional collagen gel. Furthermore, A549 cells overexpressing ATF5 showed high tumorigenic potential and development of invasive phenotypes in *in vivo* mouse experiments. In addition, ATF5 expression correlated with poor prognosis in lung cancer patients. We also investigated the molecules regulated by ATF5 and involved in development of the invasive capability of lung cancer cells. ATF5 promoted the expression of integrin β1, which is a key regulator of cell-matrix adhesion, and regulates A549 cell invasiveness. Moreover, ATF5 induced invasive phenotypes in A549 cells via prevention of the di-phosphorylation of MRLC, which drives cellular contractile forces. These findings indicate that ATF5 is a key molecule involved in lung cancer progression.

Recently, we found that ATF5 increases invasiveness in HT1080 human fibrosarcoma cells and MDA-MB-231 human breast cancer cells (unpublished data). Thus, ATF5 may contribute to the progression of various types of cancers, including lung cancer, breast cancer, and fibrosarcoma.

We demonstrated that ATF5 is crucial for radio-resistance and malignancy in lung cancer cells; however, further research is yet to be conducted to ascertain how ATF5 enhances radioresistance in lung cancer cells. One possible mechanism is by preventing senescence. Previous research shows that A549 cells are killed by senescence after irradiation [7]. Thus, ATF5 may prevent senescence following irradiation in lung cancer cells, thereby enhancing radioresistance. We are also yet to ascertain how ATF5 induces tumorigenesis in lung cancer. We showed that ATF5 triggers G1/S transition, and therefore, this transition may contribute to tumorigenic ability in lung cancer cells.

Therefore, although further research is still warranted, ATF5 is a promising therapeutic target for lung cancer. ATF5 enhances both the survival ability and malignant potential of lung cancer cells after irradiation. Thus, ATF5 inhibition can be used in two different therapeutic ways. First, inhibition of ATF5 function before radiotherapy can decrease the number of surviving cancer cells after irradiation. This method in turn may decrease the risk of tumor recurrence after radiotherapy. Second, blockage of ATF5 after radiotherapy may prevent malignant transformation of lung cancer cells that survived irradiation. In this case, even if the tumor recurs after radiotherapy, tumor progression may be perturbed. Thus, ATF5 inhibition, before and after radiotherapy, can be used as a potential therapeutic method for lung cancer treatment.
